# Single-Center Experience of Pediatric Cystic Kidney Disease and Literature Review

**DOI:** 10.3390/children11040392

**Published:** 2024-03-25

**Authors:** Sara Grlić, Viktorija Gregurović, Mislav Martinić, Maša Davidović, Ivanka Kos, Slobodan Galić, Margareta Fištrek Prlić, Ivana Vuković Brinar, Kristina Vrljičak, Lovro Lamot

**Affiliations:** 1 Department of Pediatrics, School of Medicine, University of Zagreb, 10000 Zagreb, Croatia; saragrlic2@gmail.com (S.G.); ivana.vukovic.brinar@mef.hr (I.V.B.); lovro.lamot@mef.hr (L.L.); 2 Department of Pediatrics, University Hospital Center Zagreb, 10000 Zagreb, Croatia; mislav.martinic@kbc-zagreb.hr (M.M.); masa.davidovic@kbc-zagreb.hr (M.D.); ivanka.kos@kbc-zagreb.hr (I.K.); sgalic@kbc-zagreb.hr (S.G.); kvrljica@kbc-zagreb.hr (K.V.); 3 Department of Nephrology, Arterial Hypertension, Dialysis and Transplantation, University Hospital Center Zagreb, 10000 Zagreb, Croatia; margareta.fistrek.prlic@kbc-zagreb.hr; 4 Department of Internal Medicine, School of Medicine, University of Zagreb, 10000 Zagreb, Croatia

**Keywords:** cystic kidney disease, ADPKD, ARPKD, Bardet–Biedl syndrome, Joubert syndrome, multicystic dysplastic kidney, nephronophthisis complex, tuberous sclerosis complex

## Abstract

Introduction: Pediatric cystic kidney disease (CyKD) includes conditions characterized by renal cysts. Despite extensive research in this field, there are no reliable genetics or other biomarkers to estimate the phenotypic consequences. Therefore, CyKD in children heavily relies on clinical and diagnostic testing to predict the long-term outcomes. Aim: A retrospective study aimed to provide a concise overview of this condition and analyze real-life data from a single-center pediatric CyKD cohort followed during a 12-year period. Methods and Materials: Medical records were reviewed for extensive clinical, laboratory, and radiological data, treatment approaches, and long-term outcomes. Results: During the study period, 112 patients received a diagnosis of pediatric CyKD. Male patients were more involved than female (1:0.93). Fifty-six patients had a multicystic dysplastic kidney; twenty-one of them had an autosomal dominant disorder; fifteen had an isolated renal cyst; ten had been diagnosed with autosomal recessive polycystic kidney disease; three had the tuberous sclerosis complex; two patients each had Bardet–Biedl, Joubert syndrome, and nephronophthisis; and one had been diagnosed with the trisomy 13 condition. Genetic testing was performed in 17.9% of the patients, revealing disease-causing mutations in three-quarters (75.0%) of the tested patients. The most commonly presenting symptoms were abdominal distension (21.4%), abdominal pain (15.2%), and oligohydramnios (12.5%). Recurrent urinary tract infections (UTI) were documented in one-quarter of the patients, while 20.5% of them developed hypertension during the long-term follow-up. Antibiotic prophylaxis and antihypertensive treatment were the most employed therapeutic modalities. Seventeen patients progressed to chronic kidney disease (CKD), with thirteen of them eventually reaching end-stage renal disease (ESRD). The time from the initial detection of cysts on an ultrasound (US) to the onset of CKD across the entire cohort was 59.0 (7.0–31124.0) months, whereas the duration from the detection of cysts on an US to the onset of ESRD across the whole cohort was 127.0 (33.0–141.0) months. The median follow-up duration in the cohort was 3.0 (1.0–7.0) years. The patients who progressed to ESRD had clinical symptoms at the time of initial clinical presentation. Conclusion: This study is the first large cohort of patients reported from Croatia. The most common CyKD was the multicystic dysplastic kidney disease. The most common clinical presentation was abdominal distention, abdominal pain, and oliguria. The most common long-term complications were recurrent UTIs, hypertension, CKD, and ESRD.

## 1. Introduction

Renal cysts are a frequent finding in various kidney diseases, affecting individuals of all ages, from children to adults [1]. Their complex pathological mechanisms give rise to heterogeneous clinical presentations, leading to unpredictable long-term consequences. Pediatric cystic kidney disease (CyKD) is the third most prevalent cause of end-stage renal disease (ESRD), accounting for approximately 9.7% to 14.1% of all pediatric cases needing renal replacement therapy (RRT). Despite numerous basic, translational, and clinical studies, it is still challenging to differentiate the patients who will remain asymptomatic from those who will progress to ESRD. Therefore, the current study adds to the existing literature on establishing a causal relationship between common clinical findings and progression to chronic kidney disease (CKD) [2].

Considering the paucity of data in the literature, the primary objective of our study was to analyze the clinical characteristics and outcomes of children with CyKD followed in a single tertiary referral center over a prolonged period. We conducted a retrospective analysis of all the consecutive patients during the study periods. We correlated the most common diagnoses with modifiable and non-modifiable factors like family history, genetic mutations, clinical symptoms, laboratory and imaging findings, and disease progression. We extensively reviewed the literature and formulated important considerations for the long-term follow-up of this rare group of diseases.

### 1.1. Cystic Kidney Disease as Part of the Ciliopathies Spectrum

**Figure 1:**
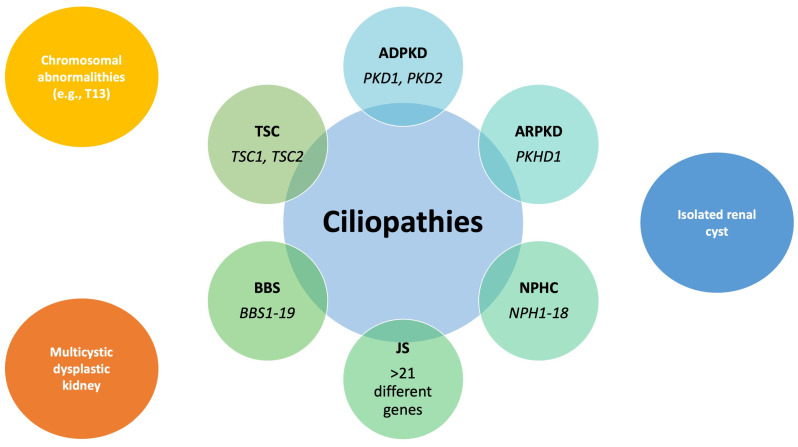
Cystic kidney disease as part of the ciliopathies’ spectrum. The name of the syndrome is written in bold and those of its most important associated genes in Italic. Abbreviations: ADPKD, autosomal dominant polycystic kidney disease; ARPKD, autosomal recessive polycystic kidney disease; BBS, Bardet–Biedl syndrome; JS, Joubert syndrome; NPHC, nephronophthisis complex; T13, trisomy 13; and TSC, tuberous sclerosis complex. Adapted from Refs. [3,9,13,14].

The term CyKD encompasses various clinical and genetic unrelated conditions characterized by “fluid-filled sacs” in the renal parenchyma (Figure 1) [3]. Recent progress in the evaluation of its underlying mechanisms has brought forth the common denominator for this entire group of diseases through the pathophysiological concept of ciliopathies [4]. These are rare disorders caused by abnormalities in the cilia, hair-like cell surface organelles responsible for the execution of many important cellular tasks. Cilia-associated disorders, commonly originating from mutations in the genes involved in cilia structure and function, can impact multiple organs and systems, leading to conditions such as polycystic kidney disease (PKD), Bardet–Biedl syndrome (BBS), nephronophthisis complex (NPHC), and Joubert syndrome (JS), with symptoms encompassing kidney dysfunction, hepatic impairment (e.g., ARPKD), vision problems, developmental delays, intellectual disabilities, and skeletal abnormalities [5,6]. Diagnosing ciliopathies can be particularly challenging due to the significant symptom overlap within the ciliopathy spectrum, the involvement of multiple associated genes, the variation in clinical manifestation, and the unpredictable timing of these diseases’ onset [6]. Nevertheless, advancements in molecular diagnostics have resulted in enhanced precision when identifying these conditions. At present, significant progress is being made in the development of pharmacological treatment strategies for ciliopathies, even though the primary approach to treatment still relies on supportive therapy [7]. Besides genetic mutations, ciliopathies can also be associated with chromosomal abnormalities, including trisomies [8,9]. For example, in some cases of trisomy 13 (T13), individuals may exhibit additional features or symptoms that are reminiscent of ciliopathy-related conditions due to the disruption of cilia-related genes or pathways caused by the chromosomal abnormality. It is not surprising that almost 50% of T13 patients exhibit kidney issues, with CyKD being the most prevalent, affecting over a third of cases. Furthermore, the tuberous sclerosis complex (TSC) is not categorized as a ciliopathy, and it is still debatable whether the malfunctioning of cilia has been linked to the development of renal cysts that associated with the TSC [10]. Henske et al. have demonstrated that loss-of-function mutations in the TSC1 or TSC2 genes can interfere with the structure and function of the cilia in renal tubular cells, thereby contributing to the formation of cysts in the kidneys [11]. Multicystic dysplastic kidney (MCDK) disorder was traditionally assumed to be a developmental anomaly in which the renal parenchyma fails to develop, leading to the presence of primitive ducts, cysts, and, potentially, tissues of extrarenal origin [12,13]. However, the research conducted by Harris et al. suggests that MCDK, in its severe form, can be classified as part of the broader spectrum of ciliopathies, requiring further research to comprehend its underlying molecular mechanisms [14].

### 1.2. A Condensed Overview of Cystic Kidney Disease(s)

Autosomal Dominant Polycystic Kidney Disease (ADPKD) is primarily caused by mutations in the PKD1 (85%) or PKD2 gene (15%), which affect the glycoproteins polycystin 1 (PC1) and polycystin 2 (PC2), respectively [15]. The timing and severity of cyst formation and symptom onset depend on the presence and nature of the gene mutation, spanning from early childhood to adulthood [16]. Given that 75–90% of ADPKD patients have a parent with the disease, it is important to inquire about the family history during diagnosis [17]. Based on recent epidemiological data (1980–2015) from the European Renal Association (ERA) and European Dialysis and Transplant Association (EDTA) registry, Willey et al. estimated a prevalence of 3.96 per 10,000 individuals (<5 per 10,000) in the general European population [18]. ADPKD typically presents between the ages of 20 and 40, but in children, it can manifest as early (<15 years), very early (<2 years), or even prenatally [1]. The natural history of ADPKD usually involves a period of stable phase followed by a gradual decline in renal function, although the progression of the disease can vary among different family members [19]. Common manifestations and complications include hypertension (HTN) (20% of children), urine concentrating defect (up to 60% of children), proteinuria, abdominal/flank pain, hematuria, nephrolithiasis, and urinary tract infections (UTIs) with high incidence (30–60%), while intracranial aneurysms (5%–9%) occur three to five times more frequently than in the general population [17,20,21,22].

Autosomal Recessive Polycystic Kidney Disease (ARPKD) is less common (1:20,000) than ADPKD and is typically diagnosed at birth through prenatal sonographic screening [23]. Infants with ARPKD often present with bilateral flank masses and a distended abdomen due to enlarged kidneys (more than four standard deviations above the average) [24]. The most common cause of ARPKD is a mutation in the PKHD1 gene, although recent studies have identified mutations in the DZIP1L gene as well [25]. In severe cases, the prenatal form can lead to oligohydramnios and pulmonary hypoplasia, resulting in respiratory distress shortly after birth [26]. Liver and biliary involvement are associated with ARPKD [27,28].

Nephronophthisis Complex (NPHC) is a condition characterized by chronic tubulo-interstitial nephritis that can progress to ESRD early in life, along with various extrarenal manifestations, depending on the mutation in NPHP1-9 genes [29,30]. Therefore, regular monitoring of kidney and liver function, along with periodic eye examinations, is crucial for patients with NPHC to ensure early detection and appropriate management.

Joubert Syndrome (JS) is a genetically heterogenous ciliopathy associated with more than 30 genes [31,32]. Though primarily characterized by nervous system symptoms such as ataxia, hypotonia, and developmental delays, renal involvement is observed in 23% to 35% of individuals with JS, often leading to ESRD in severe cases. 

Tuberous Sclerosis Complex (TSC) is a neurocutaneous condition featuring distinctive skin lesions, epileptic seizures, and abnormal growths or hamartomas in organs like the heart, brain, and kidneys [33]. Mutations in either the TSC1 or the TSC2 gene underlie the disorder [34]. Hamartin and tuberin, proteins produced by these genes, form cytoplasmic heterodimers that inhibit mTOR-mediated cell growth and division. Neurological symptoms, particularly seizures affecting around 90% of patients, are primary presentations of TSC [35]. Renal manifestations are the second most common, with approximately 80% of patients developing angiomyolipoma and 50% experiencing CyKD, particularly in cases involving TSC2 gene mutations.

Bardet-Biedl Syndrome (BBS) is a rare autosomal recessive genetic condition primarily characterized by six cardinal symptoms, including retinal dystrophy, early-onset obesity, postaxial polydactyly, intellectual disability, hypogonadism, and renal abnormalities, along with several secondary symptoms [36,37]. Around half of BBS patients exhibit various renal structural abnormalities detected by ultrasound, with renal cysts being common. A study conducted by Forsythe et al. in 2017 provides insights into the prevalence of kidney-related complications in children with BBS [38]. According to their findings, approximately 31% of children diagnosed with BBS develop CKD, with 6% progressing to ESRD. 

As reported by Goel et al., the prevalence of trisomy 13 (T13) was determined to be 1.15 per 10,000 total births, encompassing both live births and stillbirths within the studied population [39]. Clinical features include severe intellectual disability, abnormalities in the central nervous system and internal organs, polydactyly, cleft lip and palate (CLP), microphthalmia, and early mortality [9]. Around 50% of these patients tend to have anomalies of the kidneys and urinary tract, often including renal cysts. 

With a prenatal diagnosis common, Multicystic Dysplastic Kidney (MCDK) has an incidence rate of 1 in 4300 live births and only affects one kidney since the bilateral form is incompatible with life [13]. Approximately 15% of MCDK cases feature additional genitourinary tract anomalies, such as contralateral renal agenesis and vesicoureteral reflux (VUR). The affected kidney in MCDK regresses gradually, while the opposite kidney compensates by hypertrophy [12]. 

Cysts not associated with worsening kidney function and/or other abnormalities are designated as Isolated Renal Cysts [40]. They can be classified as simple or complex according to the Bosniak classification, with complex cysts being rare in children. Nevertheless, regular check-ups are important for children initially diagnosed with isolated simple renal cysts, as deterioration in renal function over time may indicate other conditions, such as ADPKD [41]. 

Lastly, other types of non-hereditary and hereditary CyKD, which are not included in this study, are complex renal cyst, cystic renal tumor, obstructive cystic dysplasia, medullary sponge kidney, glomerulocystic kidney disease (GCKD) HNF1B/TCF2-associated disease, and Von Hippel–Lindau syndrome [42]. 

## 2. Materials and Methods

### 2.1. Study Design

This was a retrospective, observational study of consecutive patients examined from July 2011 to July 2023 for CyKD in the University Hospital Center Zagreb, Department of Pediatrics, Division of nephrology, dialysis, and transplantation. 

This study site is a referral center for Pediatric Nephrology in the Republic of Croatia. Patient data was collected from computerized medical records. The tenth revision of the International Classification of Diseases (ICD-10) codes designating CyKD (Q61.0–Q61.9) was used to search the database. The digital medical records of every patient were individually reviewed by study researchers. All the patients included in the study were evaluated at least two or more times in the inpatient and/or outpatient clinic.

### 2.2. Data Collection

Researchers collected comprehensive data encompassing patients’ medical and family history, physical examination findings, and comorbid diseases. Available blood laboratory findings were analyzed, with a particular emphasis on creatinine and blood urea nitrogen (BUN) levels. 

All patients had urinalysis at initial and follow-up visits. Point-of-care urinalysis included bacteriuria, proteinuria, leukocyturia, and hematuria, pH, color, urine turbidity, specific gravity, glucose, ketones, nitrates, urobilinogen, and bilirubin. 

At least one prenatal US was performed in the entire cohort as part of standard obstetric care in the Republic of Croatia. Moreover, standard patient care involved an US examination of the kidneys in which the morphological characteristics, such as size, corticomedullary differentiation (CMD), and echogenicity were assessed by an experienced pediatric nephrologist in a binary manner. When cysts were detected, their number, location, and maximum diameter were monitored. When available, we recorded the laterality and distribution of cysts within the kidneys. The data was correlated between the first and the latest US scans. Radiological imaging techniques such as computerized tomography (CT) scans, magnetic resonance imaging (MRI), and US scans were used in selected patients to establish renal and extrarenal manifestations of CyKD.

The follow-up of patients included their age at the time of diagnosis, the specific presenting symptoms, or the time necessary for the symptoms to appear. The study also analyzed the number of hospitalizations and outpatient visits. Additionally, the study recorded the treatments provided to the patients and assessed the length of time with no medical interventions. The patients not evaluated for ≥2 years were classified as lost to follow-up.

Genetic testing was not routinely performed due to the lack of adequate public or private laboratories in Croatia, as well as consensual recommendations by professional societies. Since 2020, for limited patients with health insurance approval, samples have been tested for appropriate genetic panels in a private laboratory (Blueprint Genetics, Espoo, Finland). Karyotype was obtained in all patients with chromosomal disorders.

### 2.3. Diagnostic Criteria and Definitions

The definitive diagnosis of a CyKD variant was made by a pediatric nephrologist in patients with specific clinical features, imaging, and laboratory findings, as well as genetic testing results when available.

#### 2.3.1. Autosomal Dominant Polycystic Disease

The diagnosis of autosomal dominant polycystic disease (ADPKD) was in line with Pei Ravine’s criteria [28]. Patients older than 15 years were required to have three or more unilateral or bilateral cysts. Patients below 15 years were required to have a positive family history of nephromegaly or at least one cyst to be considered for the diagnosis of ADPKD. If the family history was unknown, parents and grandparents were required to undergo US screening. In selected patients with high suspicion who did not meet the above-mentioned clinical criteria, the diagnosis was made by genetic testing.

#### 2.3.2. Autosomal Recessive Polycystic Disease

The diagnosis of ARPKD was made according to the US findings, which included bilateral enlarged hyperechogenic kidneys, poor corticomedullary differentiation, and multiple small cysts, as well as liver involvement [28,42]. Liver disease, which included hepatomegaly, liver cysts, Caroli disease, congenital hepatic fibrosis, and signs of portal hypertension was assessed on abdominal US and, if indicated, on MRI scans. In selected patients with high suspicion who did not meet the above-mentioned clinical criteria, the diagnosis was made by genetic testing.

#### 2.3.3. Multicystic Dysplastic Kidney

The diagnosis of MCDK was established through the observation of a complete kidney replacement by disorganized cysts without normal surrounding tissue and absent or atretic ureter by the US [28,43]. If functional renal imaging was performed, it suggested minimal to no kidney function [28].

#### 2.3.4. Tuberous Sclerosis Complex

TSC diagnosis was made as described by Northrup et al. [10]. All of the patients underwent detailed clinical examination for major and minor features and the disease was confirmed by genetic testing.

#### 2.3.5. Joubert Syndrome

Diagnosis of JS was based on neuroradiologic finding of a molar tooth sign and neurologic symptom, such as hypotonia, developmental delay and dysregulation of breathing pattern, cerebellar ataxia and abnormal eye movements [44,45]. The US was used for the confirmation of renal disease and the final diagnosis was confirmed by genetic testing.

#### 2.3.6. Bardet–Biedl Syndrome

BBS diagnosis relied on clinical criteria outlined by Beales et al. necessitating a minimum of four primary features or three primary features along with two secondary features [46,47]. Clinical diagnosis was confirmed by genetic testing.

#### 2.3.7. Nephronophthisis Complex

Diagnosis of NPHC was based on US finding of normal or slightly reduced kidneys with increased echogenicity and/or combination of typical symptoms, such as polyuria, polydipsia, anemia [48,49,50,51,52,53,54,55,56]. The diagnosis was confirmed by genetic testing in all of the patients.

#### 2.3.8. Isolated Renal Cyst

The diagnosis of an IRC was made in patients with US finding of a round shape cyst appearing as a fluid-filled void without internal partitions, distinct from the collecting system, and lacking Doppler blood flow associated with the cyst, along with normal surrounding kidney tissue and a healthy opposite kidney [28].

#### 2.3.9. Recurrent UTI (rUTI), Vesicoureteral Reflux (VUR) and Antibiotic Prophylaxis

rUTI was defined as ≥2 UTI during the six months. Voiding cystourethrography (VCUG) or contrast-enhanced voiding ultrasonography (ceVUS) was performed in selected patients that had rUTI or UTI and signs of hydronephrosis on a US scan. Antibiotic prophylaxis was decided on a case-to-case basis considering rUTI, VUR grade, and other abnormalities such as hydronephrosis.

#### 2.3.10. Hypertension

The diagnosis of hypertension was made when the blood pressure (BP) measurements performed on three separate occasions were above the 95th percentile according to age, sex, and height [57].

#### 2.3.11. Proteinuria

Proteinuria in children was defined as excessive protein excretion of 100 mg/m^2^ per day or 4 mg/m^2^ per hour [58,59]. Performing a spot urine test, proteinuria was defined as a protein/creatinine ratio (Pr/Cr) of more than 0.2 mg protein/mg creatinine (>20 mg protein/mmol creatinine) in children aged more than two years and more than 0.5 mg protein/mg creatinine (>50 mg protein/mmol creatinine) in infants and toddlers from 6 to 24 months.

### 2.4. Data Analysis

The researchers obtained medical records from the electronic database of the University Hospital Center Zagreb and utilized Blueprint Genetics to access individual genetic panels. To assist in gathering medical data, a spreadsheet methodology using Microsoft Office Excel was developed. Medical data was analyzed using Jeffreys’s Amazing Statistics Programs (JASP), version 0.17.1.0. The data was described using charts and descriptive statistics and presented as raw numerical data for categories (diagnoses) that included five or fewer individuals in a category. Medians and interquartile ranges (IQR) were presented for categories (diagnoses) that included more than five individuals in a category.

### 2.5. Ethics Statement

The study was conducted per the Helsinki Declaration with the approval from the Ethics Committee of the University Hospital Center Zagreb (02/013 AG).

## 3. Results

### 3.1. Characteristics of the Patients with Cystic Kidney Disease

**Figure 2:**
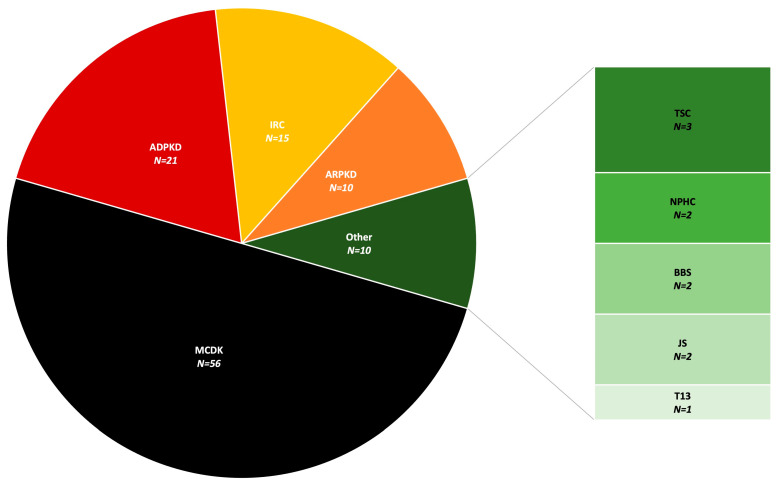
The final diagnosis of included patients. Abbreviations: ADPKD (autosomal dominant polycystic kidney disease), ARPKD (autosomal recessive polycystic kidney disease), BBS (Bardet-Biedl syndrome), IRC (isolated renal cyst), JS (Joubert syndrome), MCDK (multicystic dysplastic kidney), NPHC (nephronophthisis complex), T13 (trisomy 13), TSC (tuberous sclerosis complex).

**Figure 3:**
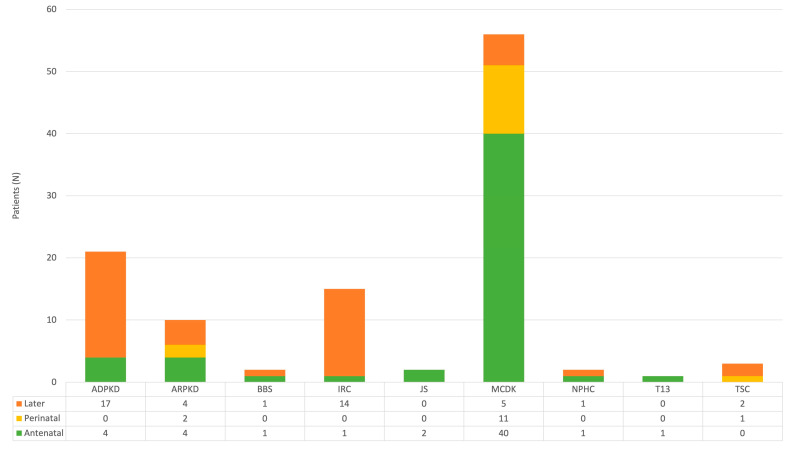
Detection of cysts by ultrasound in antenatal or perinatal (≤28 days) period, or later during childhood. Abbreviations: ADPKD (autosomal dominant polycystic kidney disease), ARPKD (autosomal recessive polycystic kidney disease), BBS (Bardet-Biedl syndrome), IRC (isolated renal cyst), JS (Joubert syndrome), MCDK (multicystic dysplastic kidney), NPHC (nephronophthisis complex), T13 (trisomy 13), TSC (tuberous sclerosis complex).

**Figure 4:**
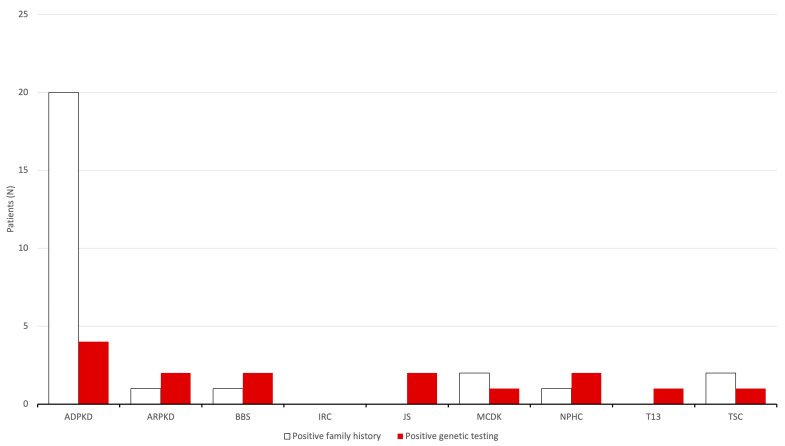
Number of patients with positive family history and genetic testing. Note that not all the patients had genetic testing. Abbreviations: (ADPKD) autosomal dominant polycystic kidney disease; (ARPKD) autosomal recessive polycystic kidney disease; (BBS) Bardet-Biedl syndrome; (IRC) isolated renal cyst; (JS) Joubert syndrome; (MCDK) multicystic dysplastic kidney; (NPHC) nephronophthisis complex; (T13) trisomy 13; (TSC) tuberous sclerosis complex.

Out of 112 patients who were included in this study, 54 (48.2%) were female and 58 (51.8%) were male. The final diagnosis was MCDK in 56, ADPKD in 21, ARPKD in 10, IRC in 15, TSC in 3, JS in 2, BBS in 2, NPHC in 2, and T13 in 1 patient (Figure 2). As part of standard obstetric care, antenatal US revealed kidney cysts in 54 patients (48.2%), while 14 (12.5%) were identified during the perinatal period (within the first 28 days) (Figure 3). An additional 44 cases (39.3%) were detected in the US later in life, specifically after the 28th day, with a median age of 6.0 (3.8–12.3) years. Among patients diagnosed with kidney cysts later in life, the median age in years was as follows: 7.0 (6.0–12.5) in the ADPKD group and 5.0 (3.3–10.8) in the IRC group. Bilateral CyKD was documented in 33 (29.5%) patients (10 ARPKD, 13 ADPKD, 3 TSC, 2 BBS, 2 JS, 2 NPHC, 1 T13). Twenty-seven (24.1%) had a positive family history of CyKD (20 ADPKD, 2 MCDK, 1 ARPKD, 2 TSC, 1 BBS, 1 NPHC) (Figure 4).

### 3.2. Presenting Symptoms and Signs of Cystic Kidney Disease

**Figure 5:**
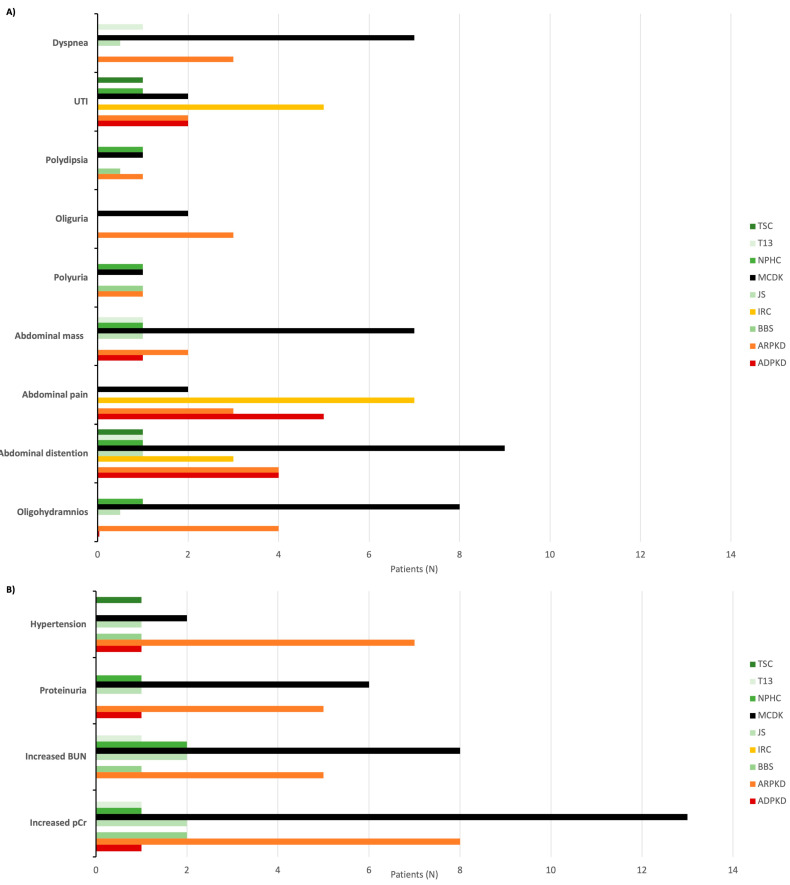
Presenting signs (**A**) and symptoms (**B**) of cystic kidney disease. Abbreviations: ADPKD (autosomal dominant polycystic kidney disease), ARPKD (autosomal recessive polycystic kidney disease), BBS (Bardet-Biedl syndrome), IRC (isolated renal cyst), JS (Joubert syndrome), MCDK (multicystic dysplastic kidney), NPHC (nephronophthisis complex), T13 (trisomy 13), TSC (tuberous sclerosis complex), UTI (urinary tract infection), BUN (blood urea nitrogen), pCr (plasma creatinine).

Oligohydramnios was observed as a prenatal presenting symptom in 14 (12.5%) children. The most frequent postnatal symptoms were abdominal distension in 24 cases (21.4%) and abdominal pain in 17 patients (15.2%). UTIs, HTN, and an abdominal palpable mass were each found in 13 cases (11.6%). At the time of diagnosis, 28 patients (25.0%) had increased plasma creatinine levels, and 19 patients (17.0%) had elevated BUN levels. Additionally, 14 patients (12.5%) presented with proteinuria (Figure 5).

### 3.3. The Renal Ultrasound Findings

**Figure 6:**
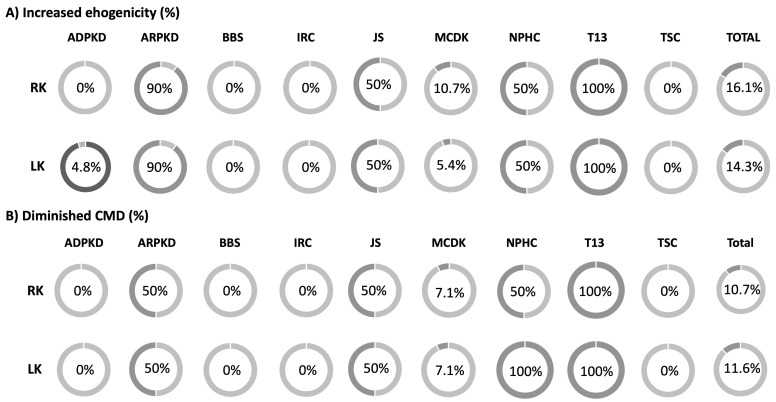
Percentage of patients with increased echogenicity (**A**) and diminished corticomedullary differentiation (CMD) (**B**) according to the diagnoses. Abbreviations: ADPKD (autosomal dominant polycystic kidney disease), ARPKD (autosomal recessive polycystic kidney disease), BBS (Bardet-Biedl syndrome), IRC (isolated renal cyst), JS (Joubert syndrome), MCDK (multicystic dysplastic kidney), NPHC (nephronophthisis complex), T13 (trisomy 13), TSC (tuberous sclerosis complex), RK (right kidney), LK (left kidney).

During the initial US examination of the kidneys increased echogenicity of the right kidney (RK) was observed in a total of 18 (16.1%) patients (9 ARPKD, 6 MCDK, 1 JS, 1 NPHC, 1 T13). Similarly, the left kidney (LK) showed increased echogenicity in 16 (14.3%) patients (9 ARPKD, 3 MCDK, 1 ADPKD, 1 JS, 1 NPHC, 1 T13). A distinctive echo pattern, formerly known as the salt and pepper pattern, was detected in a single patient with ARPKD. Diminished CMD was present in 12 (10.7%) patients on the RK (5 ARPKD, 4 MCDK, 1 JS, 1 NPHC, 1 T13) and in 13 (11.6%) patients on the LK (5 ARPKD, 4 MCDK, 2 NPHC, 1 JS, 1 T13) (Figure 6). In the group of patients with ARPKD, the first available US commonly displayed a distinctive cyst pattern, with multiple small and diffuse cysts being detected in both kidneys in 9 (90.0%) ARPKD patients. In the group of patients with ADPKD, only 5 (23.8%) had multiple cortical and medullary cysts in the RK, and 8 (38.1%) in the LK. In the ADPKD group, the more prevalent cyst pattern observed in US was the presence of a single cyst or two cysts in one or both kidneys.

### 3.4. VUR, (r)UTI and Antibiotic Prophylaxis

**Figure 7:**
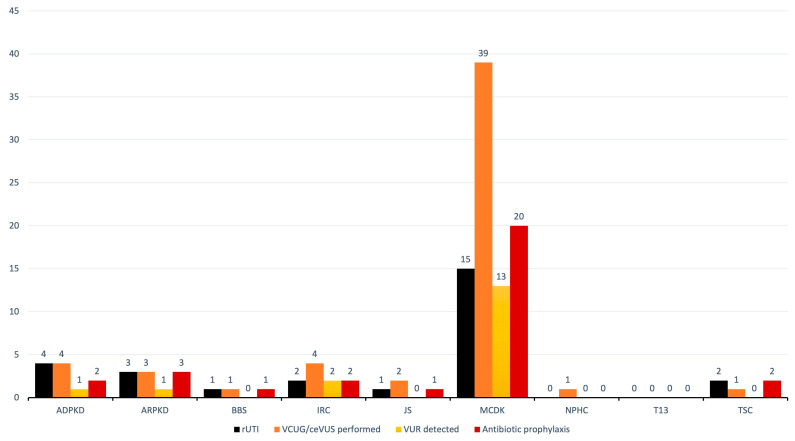
Overview of patients with rUTI, performed VCUG/ceVUS, detected VUR and employed antibiotic prophylaxis. Abbreviations: ADPKD (autosomal dominant polycystic kidney disease), ARPKD (autosomal recessive polycystic kidney disease), BBS (Bardet-Biedl syndrome), IRC (isolated renal cyst), JS (Joubert syndrome), MCDK (multicystic dysplastic kidney), NPHC (nephronophthisis complex), T13 (trisomy 13), TSC (tuberous sclerosis complex), rUTI (recurrent urinary tract infection), VCUG (voiding cystourethrography), ceVUS (contrast enhanced voiding ultrasonography), VUR (vesicoureteral reflux).

VCUG was performed in 55 (49.1%) children, with VUR detected in 17 (31.0% of the tested patients). All of them had grade II or higher VUR, except a single MCDK patient who had VUR grade I on the RK. Unilateral VUR was found in 13 (23.6%) patients (10 MCDK, 2 IRC, 1 ARPKD), whereas bilateral VUR in four (7.3%) patients (3 MCDK, 1 ADPKD). rUTI was diagnosed in 28 (25.0%) patients. The most identified causative agents of UTIs in urine cultures in order of frequency, are *K. pneumoniae*, *E. coli*, *P. mirabilis*, and *E. faecalis*. Antibiotic prophylaxis was used in 31 (27.7%) patients. Antibiotic prophylaxis included nitrofurantoin, cefixime, and cephalexin over a period of 6 to 12 months. An overview of the VUR incidence and administered antibiotic prophylaxis is shown in Figure 7.

### 3.5. Genetic Testing

Genetic testing was performed on 20 patients in total (17.9%) and was positive for a disease-causing mutation in 15 (75.0% of tested patients) (Figure 4). A mutation in the PKD1 gene was detected in one patient with ADPKD and one patient with TSC. The patient with TSC also had an accompanying mutation in the TSC2 gene, causing a TSC2/PKD1 contiguous gene syndrome (TSC2/PKD1-CGS). A mutation in the PKD2 gene was identified in three siblings, although only one of them had developed symptoms at the time of the genetic testing. The mutation in the PKHD1 gene was found in two patients with ARPKD, while one of them had an additional likely pathogenic mutation in the PTPN11 gene. The CEP290 gene mutation was present in two patients with JS and one patient with NPHC. Additionally, two patients with BBS had a mutation in the BBS12 gene, with one of them having a variant of uncertain significance (VUS) in the BBS1 and BBS5 genes. A mutation in the NPHP1 gene was present in one patient with NPHC. A probable pathogenic deletion (1p36.12–p36.11) was documented in a single patient with MCDK, whereas the patient with T13 had the Robertsonian translocation rob (13;14) and was diagnosed with bilateral polycystic kidneys antenatally.

### 3.6. Progression to Chronic Kidney Disease (CKD), End-Stage Renal Disease (ESRD), and Renal Replacement Therapy (RRT)

**Figure 8:**
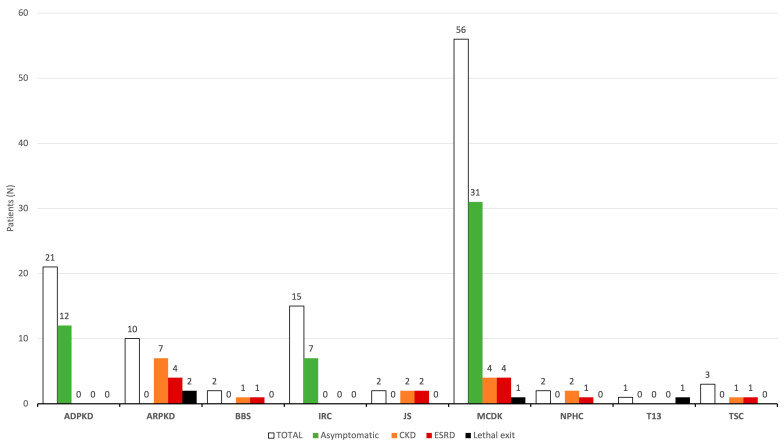
Outcome of study patients during the follow up. Abbreviations: ADPKD (autosomal dominant polycystic kidney disease), ARPKD (autosomal recessive polycystic kidney disease), BBS (Bardet-Biedl syndrome), IRC (isolated renal cyst), JS (Joubert syndrome), MCDK (multicystic dysplastic kidney), NPHC (nephronophthisis complex), T13 (trisomy 13), TSC (tuberous sclerosis complex), CKD (chronic kidney disease), ESRD (end-stage renal disease).

Progression to CKD occurred in 17 (15.2%) patients (7 with ARPKD, 4 with MCDK, 2 with JS, 2 with NPHC, 1 with BBS, 1 with TSC), with 13 (76.5%) out of 17 CKD patients developing ESRD (4 with ARPKD, 4 with MCDK, 2 with JS, 1 with BBS, 1 with NPHC, 1 with TSC) (Figure 8). The time from the initial detection of cysts on ultrasound to the onset of CKD across the entire cohort was 59.0 (7.0–124.0) months, whereas the median time for the development of CKD in the ARPKD patients was 124.0 (35.5–159.0) months. The duration from the detection of cysts on ultrasound to the onset of ESRD within the whole cohort was 127.0 (33.0–141.0) months. Regarding RRT, peritoneal dialysis (PD) emerged as the most common option utilized in seven patients. Additionally, five patients required continuous renal replacement therapy (CRRT) at some point. Finally, renal transplantation (RT) was performed in six patients. 

### 3.7. Follow-Up of Patients

During the follow-up, four patients died (2 ARPKD, 1 MCDK, 1 T13) and eight were transferred to adult care in a coordinated process of transition (3 ADPKD, 3 ARPKD, 1 MCDK, 1 TSC). Out of 112 patients, 45 (40.2%) were lost to follow-up. The median follow-up duration in the cohort was 3.0 (1.0–7.0) years. The duration of care without medical interventions, which includes administration of any medication for symptoms and signs related to CyKD, was 2.0 (0.0–5.3) years. Out of the total 112 patients, 50 (44.6%) remained asymptomatic during the follow-up. An overview of the follow-up data is presented in Table 1.

### 3.8. Administered Medications and Surgical Interventions during the Follow-Up

**Figure 9:**
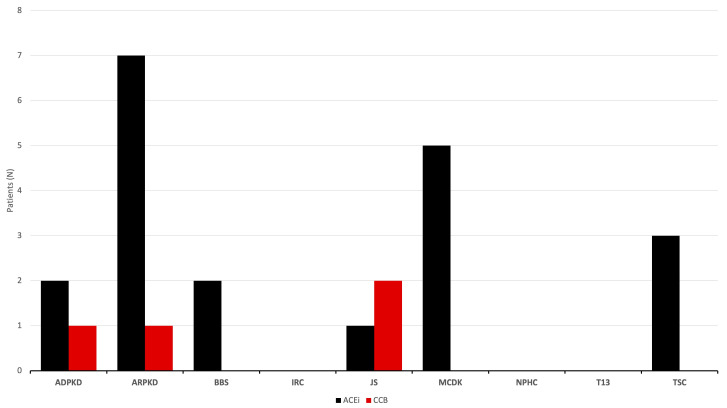
Antyhipertensive medications according to diagnosis. Abbreviations: ADPKD (autosomal dominant polycystic kidney disease), ARPKD (autosomal recessive polycystic kidney disease), BBS (Bardet-Biedl syndrome), IRC (isolated renal cyst), JS (Joubert syndrome), MCDK (multicystic dysplastic kidney), NPHC (nephronophthisis complex), T13 (trisomy 13), TSC (tuberous sclerosis complex), ACEi (angiotensin-converting enzyme inhibitor), CCB (calcium channel blocker).

Commonly administered medication during follow-up was an angiotensin-converting enzyme inhibitor (ACEi) administered to 20 (17.9%) patients, while calcium-channel blocker (CCB) was administered to 4 (3.8%) patients (one patient with JS was simultaneously administered ACEi and CCB) (Figure 9). Everolimus treatment was initiated in two patients with TSC. All TSC patients received anticonvulsant medications, including vigabatrin, sodium valproate, and carbamazepine, respectively. Finally, unilateral nephrectomy was performed in 7 MCDK (12.5% of MCDK) patients, of which partial nephrectomy in one and complete unilateral nephrectomy in 6. Additionally, a complete unilateral nephrectomy was performed in 1 ARPKD patient.

### 3.9. Extrarenal Cysts and Manifestations

**Figure 10:**
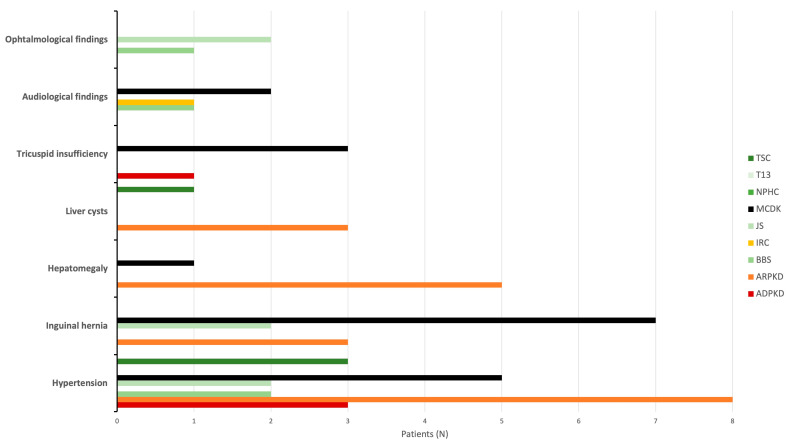
Extrarenal manifestations according to diagnosis. Abbreviations: ADPKD (autosomal dominant polycystic kidney disease), ARPKD (autosomal recessive polycystic kidney disease), BBS (Bardet-Biedl syndrome), IRC (isolated renal cyst), JS (Joubert syndrome), MCDK (multicystic dysplastic kidney), NPHC (nephronophthisis complex), T13 (trisomy 13), TSC (tuberous sclerosis complex).

The most frequently encountered extrarenal manifestations of CyKD in the study cohort were hypertension (HTN), inguinal hernia, hepatomegaly, liver cysts, tricuspid insufficiency, and various ophthalmological findings (Figure 10). HTN was present in a total of 23 (20.5%) patients during the follow-up period (8 ARPKD, 5 MCDK, 3 ADPKD, 3 TSC, 2 BBS, 2 JS). Unilateral inguinal hernia was diagnosed in 12 (10.7%) patients (7 MCDK, 3 ARPKD, 2 JS). Hepatomegaly was noted in a total of six (5.4%) children (5 ARPKD, 1 MCDK). In addition, liver cysts were identified in four (3.6%) patients (3 ARPKD, 1 TSC). Caroli’s disease was documented in a single patient with ARPKD. Splenomegaly was identified in two patients within the cohort, both of whom were diagnosed with ARPKD. Tricuspid insufficiency was observed in four (3.6%) patients (3 MCDK, 1 ADPKD). Audiological findings were identified in four (3.6%) patients. Among them, two individuals with MCDK, one with BBS, and one with IRC exhibited bilateral conductive hearing loss attributed to chronic otitis media. Furthermore, one of the patients with MCDK experienced an additional sensorineural hearing impairment. Ophthalmological findings were identified in 3 (2.7%) children within the cohort. These findings included retinitis pigmentosa (1 BBS, 1 JS) and Leber congenital amaurosis (1 JS). Lastly, all three patients diagnosed with TSC were found to have cortical tubers and subependymal nodules. None of the subependymal nodules showed signs of transformation into subependymal giant cell astrocytoma (SEGA). Additionally, one patient had arachnoid cysts detected on an MRI brain scan, while another patient had cardiac rhabdomyoma and a liver lipoma.

## 4. Discussion

Although CyKD is not common, renal cysts could be found in children accidentally or due to the presenting symptoms accountable to the renal disease (1). The finding of a cyst by a renal US in children is rarer than in adults but often causes anxiety in patients and their parents, and sometimes in inexperienced physicians.

There is ambiguity in predicting the contribution of the cyst(s) to overall health and long-term renal outcomes [60]. Although the identification of the causative genetic mutation(s) can help with the better characterization of the cysts, genotype-phenotype correlation is often heterogeneous. Genetic testing is still far from being a part of a regular clinical workup, both in developed and developing countries [4,61,62,63,64,65]. Therefore, describing the clinical and laboratory findings and outcomes in patients with CyKD can still provide important and valuable information for clinical practice [66]. Nevertheless, due to population genetic and environmental epigenetic influences, single-center cohort studies cannot be generalized [67].

### 4.1. Ultrasound

Most of the patients in our cohort had a presenting symptom or sign, ranging from UTI to abnormal laboratory results and a positive family history, which triggered evaluation with a renal US as a basic method to begin a diagnostic process. Importantly, the 2019 International Working Group Consensus Statement unambiguously supported the utilization of the US as the primary imaging approach for the evaluation of kidney cysts in pediatric patients [28]. We could easily say that the renal US represents a “window to kidney and urinary tract”, which delineates further evaluation. In the case of kidney cysts, the clinical evaluation includes urinalysis, kidney function tests, and other systems imaging. Even with enormous advances in genetic testing, which recently became readily available in numerous centers, the detailed clinical characterization of the cysts and kidney characteristics in the US still represents one of the most important steps of the evaluation. CyKD can be suspected already in the antenatal US if there is the presence of oligohydramnios, a non-visualized bladder, hyperechoic and/or enlarged kidneys, lung hypoplasia, and other congenital malformations [28]. In our study, an antenatal diagnosis of CyKD was made in nearly half of the patients based on prenatal US examinations. The importance of prenatal US is further emphasized with the example of MCDK, which was the most common form of CyKD in our cohort, present in half of the patients, with about three quarters diagnosed during the prenatal period, similar to the study of Kopač et al. [67]. Prenatal diagnosis of MCDK also probably contributes to the fact that the majority of these children are seen by pediatric nephrologists immediately after birth [67]. Comparable to the study of Kopač et al., MCDK in our cohort was more prevalent in male patients (57.69%), which is a common but not well studied association [67]. Even though renal US can differentiate different cysts, sometimes the diagnosis is ambiguous. Presently, commercially available genetic testing helps complement an accurate diagnosis [28,68].

### 4.2. Hypertension

Large population studies have found that children with ADPKD often have masked, isolated nocturnal, or non-dipping HTN, which is a nighttime decrease in BP of less than 10% compared to daytime values [69,70,71]. Hence, diagnosis of HTN in ADPKD should require not only home and clinic measurements but 24 h ambulatory BP measurement (ABPM) as well [71]. In our ADPKD cohort, 14.3% of patients were diagnosed with HTN during the follow-up period, but Marlais et al.’s study, reported an approximate 20% with HTN in their ADPKD cohort [20]. Among the ARPKD in our cohort, 80.0% of patients were diagnosed with HTN during the disease course. Most patients are diagnosed with HTN within the first few months of life, emphasizing the urgent need for referral, close monitoring, and effective management [17]. Therefore, it remains crucial to correctly diagnose HTN in pediatric CyKD [72]. The prevalence of HTN in patients with TSC who are under the age of 25 can be as high as 25% [73]. Similarly, one-third of patients with BBS may experience HTN [37]. In our study, all the individuals in the BBS, TSC, and JS groups had HTN. Among patients with TSC, there is a notable association between HTN and mutations in the TSC2 gene, especially in those diagnosed with TSC2/PKD1-CGS [73,74,75]. Approximately 2–5% of patients harbor combination mutations TSC2/PKD1 [76]. In our cohort, a single patient had TSC2/PKD1-CGS and, at 7 months, had epilepsy. Further testing showed polycystic kidneys, suggestive of early onset ADPKD and HTN. Studies by Cadnapaphornchai et al. strengthened the evidence of using ACEi in pediatric CyKD with borderline HTN, stabilized renal function, and left ventricular mass index [77]. In our cohort, 20 patients had HTN and received ACEi as an antihypertensive.

### 4.3. UTIs

Fifty percent of rUTIs are associated with congenital anomalies of the kidneys and urinary tract (CAKUT) [78]. The possible causal factors associated with UTIs in CyKD are still a subject of debate, between the ascending infection and the cyst infection [79,80]. In our cohort, we observed that UTI was present as an initial symptom in 13 children (11.6%), while rUTI was observed in a quarter of patients during the follow-up. Interestingly, 15.2% of all patients had VUR. Since the prevention of rUTI is a critical aspect of medical management in children with CyKD, we had a low threshold to start antibiotic prophylaxis in our CyKD cohort. Consequently, one or more courses of antibiotic uroprophylaxis were used in 31 children (27.7%) who had rUTI, VUR, and/or other malformations such as hydronephrosis.

### 4.4. Genetic Testing

There is a continuing debate on genetic screening in children who are at risk for ADPKD with normal renal US, or even those who have obvious US findings [17,81,82]. According to KDIGO, the current recommendation does not support routine screening for ADPKD in asymptomatic children [83]. There is still limited evidence to indicate that confirming a diagnosis of ADPKD in children will change the course of the disease or yield improved outcomes [81]. Furthermore, genetic testing for ADPKD is often expensive, and healthcare insurance providers in certain countries may not cover the costs of testing for patients without symptoms. This further reinforces the recommendation against routine screening for children. It is worth noting that recommendations may vary depending on individual circumstances, and it is advisable to consult with genetic counselors for personalized guidance. In our case series, two asymptomatic siblings, without US cysts, underwent genetic testing due to their positive family history (the mother had an ADPKD with a lethal outcome) and had the mutation of the PKD2 gene. Besides, a probable pathogenic deletion (1p36.12p36.11) was documented in a single patient with MCDK. There is limited evidence to support the significance of this deletion in the formation of cysts. However, loss of heterozygosity for this allele increases the risk of death and relapse in patients with Wilms tumor, and a similar deletion was associated with a patient with an oral cleft [84,85]. Two patients with JS and one with NPHC had the mutation in the CEP290 gene. The frequency of these gene mutations in JS patients with cerebello-oculo-renal syndrome (CORS), a subgroup of JS, is approximately 50% [86]. However, only a small proportion (around 20%) of *CEP290* mutations are responsible for other subgroups of JS. Mutation in the *CEP290* gene is a significant factor contributing to retinal dystrophy or congenital blindness, with all *CEP290* mutations showing some degree of retinopathy [45]. In our cohort, we identified one JS patient affected by retinal dystrophy, while another JS patient presented with Leber congenital amaurosis, a condition attributed to *CEP290* gene mutations in approximately 30% of cases [87]. The renal disease associated with CEP290 mutations is mainly characterized by juvenile NPHC, with the onset of renal failure typically occurring during the first or second decade of life. However, the age at which renal failure occurs can vary, with some patients developing the infantile form of NPHC, which rapidly progresses to renal failure within the first few years of life [88]. Three patients with CEP290 mutation in our cohort behaved like infantile form, progressed rapidly to CKD within three, seven and 15 months, respectively.

Genetic mutations could be hereditary or de novo, which can be differentiated most commonly by a detailed family history and/or genetic testing. Structural or functional changes of the protein could be at the level of transcription, translations, or post-translations protein modification. Complete molecular mechanisms encompassing the structure and function of protein are difficult to identify from a single panel of genetic testing. Therefore, structural and functional protein studies may provide better evidence when the genetic mutation is not identified in standard testing [89,90,91]. Genetic testing would help plan or anticipate the natural course of illness, as in the present study CEP 290 mutation would have guided the clinicians and families for early renal replacement therapy or enrolling in renal transplant lists.

Genetic testing and counseling help in anticipating the penetrance of genetic defects to the offspring [92,93]. Genetic testing of both partners who have a positive family history and anticipating the risk to offspring helps reduce the burden of hereditable CyKD in the community. In eligible families, preimplantation genetic testing for monogenic kidney disease (PGT-M) can be readily offered if available [94,95,96]. At present, due to a lack of readily available expertise and limited financial budgeting, the above testing and procedures are limited to a few centers.

### 4.5. Progression to CKD, ESRD and Follow-Up of Patients

None of the patients in the ADPKD cohort had biochemical evidence of renal impairment, and they did not progress to ESRD, similar to the study by McEwan et al. [97]. A significant proportion of patients, i.e., 11 out of 21 (52.4%) of these patients, were lost to follow-up, possibly due to false assurance of normal renal function and lack of symptoms [98]. Remarkably, ensuring the importance of lifestyle modifications and regular examinations may prevent the rapid progression of disease [71].

It is suggested to maintain optimal body weight by limiting consumption of sugary and highly processed foods, increasing fluid intake and regular physical activity, and adhering to a low-sodium diet [99]. The disease progression of ADPKD can be monitored with serial beta-2 microglobulin (β2MG) and monocyte chemotactic protein-1 (MCP-1) [100,101]. Studies in adults showed an inverse correlation between kidney volume measured by MRI or three-dimensional ultrasound (3DUS) and kidney function [102,103,104]. Cystatin C, a marker used as an early indicator of kidney dysfunction in some centers, was measured only in a few of our patients. As recently explained in an editorial by Vidal et al., the definition of neonatal acute kidney injury (AKI) by KDIGO (Kidney Disease Improving Global Outcomes), RIFLE (an acronym for Risk-Injury-Failure-Loss-End stage kidney), and AKIN (Acute Kidney Injury Network) still heavily relies on serum creatinine [105].

In our ARPKD cohort, seven patients (70.0%) developed CKD and four patients (57.1%) further progressed to ESRD. The median time for progression to CKD in our ARPKD patients was 124.0 (35.5–159.0) months [17]. For two of our ARPKD patients, the disease had a lethal outcome. One of them had a severe clinical presentation immediately after birth, characterized by multiple cardiac malformations and pulmonary hypoplasia. The other patient was prematurely born, suffered from intrauterine growth restriction (IUGR), oligohydramnios, electrolyte abnormalities (hyponatremia), pulmonary hypoplasia, and extremely enlarged kidneys that compressed other organs in the abdominal cavity. He underwent nephrectomy, and PD was initiated, but due to chronic respiratory insufficiency, he died at the age of four months.

One child with MCDK from our cohort also succumbed to the disease, as she also had chromosome 22 partial tetrasomy, also known as cat eye syndrome (CES). CES presents with ocular coloboma, congenital heart defects, anal atresia with a fistula, renal malformations such as unilateral absence, unilateral or bilateral hypoplasia, and cystic dysplasia [106]. Of the remaining MCDK patients in our cohort, four (7.1%) developed CKD, and eventually progressed to ESRD. MCDK is predominantly a unilateral disease, the other kidney undergoes hypertrophy, and very few progress to CKD/ESRD. In our cohort of MCDK with ESRD, the possible explanations for their progression are hypoplasia of the contralateral kidney in one patient, ureteral stenosis of the contralateral kidney in another patient, and the two remaining presented with contralateral VUR and posterior urethral valve (PUV) [67,107].

### 4.6. Limitations of the Study

Our study has limitations as this was a retrospective analysis, which comes with its shortfalls. Another limitation was the small sample size. The electronic medical records are limited to one center, limiting access to data from another center. A significant proportion of patients were lost to follow-up. A few patients also had a short period of follow-up. Very few patients had genetic testing. This study heavily relied on radiology for a definite diagnosis, possibly leading to a misclassification of a few patients [23,108]. The above limitations make it hard to generalize the present study outcomes.

## 5. Conclusions

**Figure 11:**
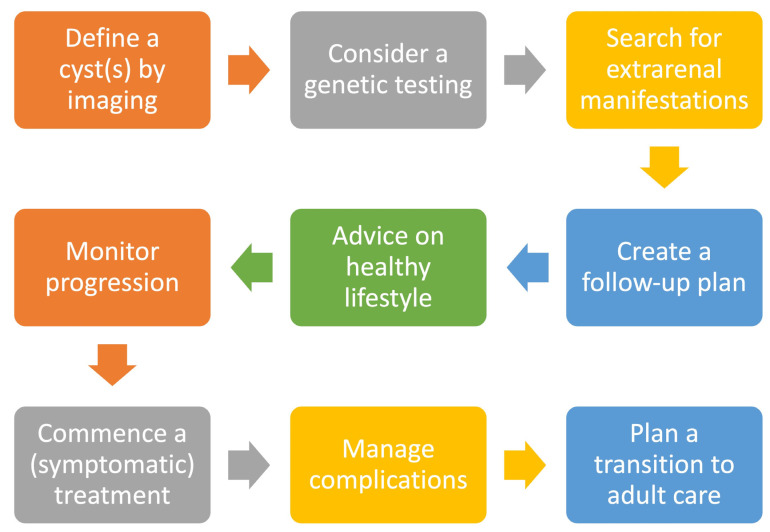
Important steps in the long-term management of children with CyKD.

This study is the first large cohort of patients reported from Croatia. The most common CyKD is multicystic dysplastic kidney (MCDK). The most common clinical presentation is abdominal distention, abdominal pain, and oliguria. The most common long-term complications are recurrent UTI, hypertension, CKD, and ESRD. The analyzed data along with literature review might serve as a signpost when seeing a new renal cyst in clinical settings (Figure 11, Table 2).

## Figures and Tables

**Table 1:** Follow-up of patients with cystic kidney disease.

Diagnosis	ADPKD	ARPKD	BBS	IRC	JS	MCDK	NPHC	T13	TSC	Total
Patients (N, %)	21 18.8%	10 8.9%	2 1.8%	15 13.4%	2 1.8%	56 50.0%	2 1.8%	1 0.9%	3 2.7%	112 100.0%
Lost to follow-up (N, %)	11 52.4%	2 20.0%	0	8 53.3%	0	23 41.1%	1 50.0%	0	0	45 40.2%
Transition to adult care (N, %)	3 14.3%	3 30.0%	0	0	0	1 1.8%	0	0	1 33.3%	8 7.1%
Follow-up in years (median (IQR))	3.0 (1.5–5.0)	4.0 (2.0–8.0)	-	3.0 (1.0–6.5)	-	3.0 (1.0–6.3)	-	-	-	3.0 (1.0–7.0)
Years without medications or medical interventions (median (IQR))	3.0 (1.0–5.0)	0.0 (0.0–1.8)	-	2.0 (1.0–6.5)	-	2.5 (1.0–6.3)	-	-	-	2.0 (0.0–5.3)
Number of ambulant visits (median (IQR))	4.0 (2.0–8.5)	7.5 (1.0–25.0)	-	5.0 (2.0–8.5)	-	6.0 (1.0–12.8)	-	-	-	5.0 (2.0–14.0)
Number of hospitalizations (median (IQR))	0.0 (0.0–1.0)	2.0 (1.0–5.0)	-	0.0 (0.0–1.0)	-	1.0 (1.0–2.0)	-	-	-	1.0 (0.0–2.0)

Abbreviations: ADPKD (Autosomal Dominant Polycystic Kidney Disease), ARPKD (Autosomal Recessive Polycystic Kidney Disease), BBS (Bardet-Biedl Syndrome), IRC (Isolated Renal Cyst), JS (Joubert Syndrome), MCDK (Multicystic Dysplastic Kidney), NPHC (Nephronophthisis Complex), T13 (Trisomy 13), TSC (Tuberous Sclerosis Complex), ACEi (Angiotensin-Converting Enzyme Inhibitor), and CCB (Calcium Channel Blocker).

**Table 2:** Important considerations for the long-term management of children with CyKD.

**Growth and development monitoring**	**Serial monitor height, weight, and developmental milestones as per available national guidelines.** **If CKD has developed or a syndrome (e.g., BBS) has been recognized adhere to specific guidelines [109,110].**
**Screening for hypertension**	Serial monitoring blood pressure (BP) at least once a year in patients with ADPKD (might be applicable for other CyKD, except IRC) [28]. Prefer ambulatory BP monitoring [111]. If hypertension has developed adhere to specific guidelines [112].
**Antihypertensive medication**	Consider starting antihypertensive medications at a lower antihypertensive treatment threshold (ninetieth percentile for age, sex, and height, which equals 130/85 mmHg on clinic measurements for those ≥16 years of age) if CKD has developed [113]. Prefer ACEi or ARB [114]. Consider aggressive blood pressure control (24 h mean arterial blood pressure below the 50th percentile for age, height, and sex) [115].
**Decreasing the risk for UTI**	Perform urinalysis and cultures when UTI is suspected [116]. Consider VCUG and DMSA kidney scans after the first febrile UTI, especially in patients with MCDK [117,118]. Consider antimicrobial prophylaxis, especially with BBD [119,120]. Consider surgical intervention with VUR [121].
**Follow-up renal ultrasound**	ADPKD: consider only if there a new clinical events (UTIs, hematuria, abdominal pain) [98]. ARPKD: consider prenatally every 2–3 weeks for serial assessment of the renal size and amniotic fluid volume, confirm diagnosis postnatally, and perform abdominal ultrasound at the age of 5 years [114]. BBS: consider yearly screening for renal tract malformations (e.g., dysplasia, agenesis, cysts, scarring) [110]. IRC: consider at least one follow-up US to rule out the development of another CyKD [28]. MCDK: consider serial measurements with defined intervals to ensure adequate compensatory hypertrophy of the contralateral kidney [118]. JS: consider periodic evaluation during the abdominal ultrasounds performed to follow spleen size [122]. TSC: consider US follow-up of renal lesions every 1–2 years [123].
**Renal function monitoring**	Consider urine ACR every 1–2 years in patients with ADPKD (might be applicable for other CyKD, except IRC) [124]. Consider creatinine, BUN and cystatin C when available at least once a year in patients with BBS (might be applicable for other CyKD, except IRC) [110]. If CKD has developed, adhere to specific recommendations [83].
**Weight and lifestyle management**	Consider dietary sodium (salt) restriction, increased fluid intake, no protein restriction, and maintaining normal weight in patients with ADPKD (might be applicable for other CyKD, except IRC) [125]. To optimize weight gain and growth in patients with ARPKD provide aggressive nutritional intervention, including supplementary feedings [126]. Consider weight management with exercise and diet in patients with BBS [47,127]. If CKD has developed, adhere to specific recommendations [128].

Abbreviations: ADPKD, autosomal dominant polycystic kidney disease; ARPKD, autosomal recessive polycystic kidney disease; BBS, Bardet–Biedl syndrome; IRC, isolated renal cyst; JS, Joubert syndrome; MCDK, multicystic dysplastic kidney; NPHC, nephronophthisis complex; T13, trisomy 13; TSC, tuberous sclerosis complex; CyKD, cystic kidney disease; CKD, chronic kidney disease; BP, blood pressure; ACEi, angiotensin-converting enzyme inhibitor; ARB, angiotensin receptor blocker; UTI, urinary tract infection; VCUG, voiding cystourethrography; DMSA kidney scan, dimercaptosuccinic acid kidney scan; BBD, bladder bowel disfunction; VUR, vesicoureteral reflux; US, ultrasound; ACR, albumin creatinine ratio; BUN, blood urea nitrogen.

## Data Availability

The data presented in this study are available on request from the corresponding author. Due to the protection of personal data, the data are not publicly available.
